# Supplementary material for: Clinical questions in primary care: Where to find the answers - a cross-sectional study
Source: PLoS One. 2022 Nov 11;17(11):e0277462. doi: 10.1371/journal.pone.0277462 (PMC9651552; doi:10.1371/journal.pone.0277462)
Supplement: Attachment — Submitted filename: Response to Reviewers.docx [file pone.0277462.s001.docx]

We hereby respond to each point raised by the reviewer:

1. Is the manuscript technically sound, and do the data support the conclusions?

The manuscript must describe a technically sound piece of scientific research with data that supports the conclusions. Experiments must have been conducted rigorously, with appropriate controls, replication, and sample sizes. The conclusions must be drawn appropriately based on the data presented.

Reviewer #1: Yes

2. Has the statistical analysis been performed appropriately and rigorously?

Reviewer #1: Yes

3. Have the authors made all data underlying the findings in their manuscript fully available?

The [PLOS Data policy](http://www.plosone.org/static/policies.action#sharing) requires authors to make all data underlying the findings described in their manuscript fully available without restriction, with rare exception (please refer to the Data Availability Statement in the manuscript PDF file). The data should be provided as part of the manuscript or its supporting information, or deposited to a public repository. For example, in addition to summary statistics, the data points behind means, medians and variance measures should be available. If there are restrictions on publicly sharing data—e.g. participant privacy or use of data from a third party—those must be specified.

Reviewer #1: No

**Thank you for your comment. Our data will be upload to a public repository (Zenodo), as soon as we have access to the publication date, which is needed for uploading datasets in this platform.**

4. Is the manuscript presented in an intelligible fashion and written in standard English?

PLOS ONE does not copyedit accepted manuscripts, so the language in submitted articles must be clear, correct, and unambiguous. Any typographical or grammatical errors should be corrected at revision, so please note any specific errors here.

Reviewer #1: Yes

5. Review Comments to the Author

Please use the space provided to explain your answers to the questions above. You may also include additional comments for the author, including concerns about dual publication, research ethics, or publication ethics. (Please upload your review as an attachment if it exceeds 20,000 characters)

Reviewer #1: The authors collected 238 clinical questions that primary care physicians raised in ambulatory care and assessed whether these questions could be answered by evidence-based resources. They found that over 92% of the questions could be answered, primarily using clinical summaries, and in a relatively short time. The manuscript is well-written and the study adds to the knowledge about the potential utility and feasibility of using evidence-based resources in patient care. Suggestions to improve the manuscript are provided below.

1) Consider adding the search time measure to the abstract, since search time is critical to determine feasibility of pursuing those questions at the point-of-care.

**Thank you for your suggestion. We added the search time measure to the abstract.**

2) Readers may not know the difference between “background” and “foreground” questions, so it would be important to define in the methods.

**Thank you for your suggestion. We added the definition of “background” and “foreground” questions in the methods section.**

3) In the results, the following statement is ambiguous: “no question was answered by systematic review synopses (level 4) and no question was answered by primary studies synopses (level 5)”. Were systematic reviews/primary studies actually searched for every single question? I assume that, based on the study methods, they were not. But it would be important to make it more clear.

**Thank you for your comment. We clarified the statement to “most clinical questions (187 of 206, 90.8% CI 95% 85.9%-94.4%) were successfully answered using level 2 resources (clinical summaries, namely, BMJ Best Practice, DynaMed or UpToDate). Three questions were answered using guidelines (level 3). Within the 30-minute time limit, no question was answered by systematic review synopses (level 4) or primary studies synopses (level 5), but one answer was found in a primary study (non-previously appraised).”**

4) As proposed above, the search time outcome is critical. Rather than just median and range, consider adding the entire distribution, perhaps including a histogram or a table. This could also have implications for the discussion. For example, even if most questions could be answered in less than 90 seconds, a relatively small percentage of questions taking longer than 5 minutes could be sufficient to discourage physicians who may be very concerned about pursuing an answer without knowing upfront how long it could take.

**Thank you for your suggestion. We added a histogram representing the entire search time distribution in the results section.**

**Figure 1. Search time distribution.** Mean search time (2 researchers), in minutes

**Regarding the implications of these findings, we added the following paragraph to the discussion section:**

**“Although the majority (67,9%) of the questions was answered in less than 5 minutes, there was still a considerable amount of questions that took more than 5 minutes to answer, which could decrease the feasibility of this method. The mean search time of researcher 2 was significantly shorter than researcher 1, who was less experienced in evidence-based practice, and the mean search time decreased in the second half of questions for both researchers. This exploratory analysis on the researchers’ search time suggested that practicing searching for evidence-based answers decreases the time needed for the searches. This is consistent with a recent systematic review that suggests search time can be optimized with practice. However, since this was not a main outcome of the study, these results should be interpreted with caution.”**

5) In the discussion, 4th paragraph, another potential nudge is “infobuttons” that not only bypass authentication but also automate the search against a set of pre-defined resources.

**Thank you for your suggestion. We added this information in the discussion.**

6) Was there any effort to validate that the answers found were sufficient to inform decision-making and supported by high-quality evidence? It appears that this determination (found vs. not found) was based on the subjective assessment of the co-authors who conducted the searches and were not blinded to the study objectives. This weakness is arguably more important than any of the limitations discussed in the manuscript.

**Thank you for your comment, and for the identification of a relevant limitation that prompted us to conduct a sensitivity analysis. We asked two blinded medical assessors who were not part of the research team to evaluate if the answers found by the researchers were considered adequate to inform decision-making. We then performed a conservative analysis, considering any answer classified by one of the blinded outcome assessors as “not informative” as an answer not found in the available resources, and only the answers classified by both assessors as “informative” as answers found in the available resources. Contrary to our expectation, this sensitivity analysis yielded a more favorable result than the primary analysis (sensitivity analysis: 198 of the 206 answers considered informative for decision-making by both blinded medical assessors [96.1%, CI 95% 92.5%-98.3%]; primary analysis: 191 of the 206 answers considered informative for decision-making by the researchers [92.7%, CI 95% 88.3%-95.9%]).**

**Regarding the quality of evidence, we registered the strength of the recommendation behind each answer, rated by clinical summaries and guidelines, and presented it according to the Grading of Recommendations Assessment, Development and Evaluation (GRADE) approach as strong, weak/conditional, or not classified. Considering the 74 foreground questions, 11 were answered with strong recommendations, 18 were answered with conditional/weak recommendations, 21 were not classified by the resource and 24 were considered not applicable to this classification (due to multiple recommendations within the same answer).**

**We added these analyses to the manuscript.**
