# Supplementary material for: Clinical questions in primary care: Where to find the answers - a cross-sectional study
Source: PLoS One. 2022 Nov 11;17(11):e0277462. doi: 10.1371/journal.pone.0277462 (PMC9651552; doi:10.1371/journal.pone.0277462)
Supplement: Attachment — Submitted filename: Response to Reviewers.docx [file pone.0277462.s002.docx]

We hereby respond to the point raised by reviewer #2 and add a correction to the reference list.

**Review Comments to the Author**

Reviewer #2: Table 1 needs a more descriptive legend. Please describe each column, especially the 'Experience' column.

**Thank you for your comment. We clarified the legend with a description of each column.**

**Table 1. Age and experience distribution. Participants’ age was reported in 10-year categories to maintain anonymity, as well as clinical experience (years practicing with autonomy); the number of participants in each age and experience category is registered under frequency (total sample = 31).**

| **Age category (years)** | **Frequency** | **Experience category (years)** | **Frequency** |
| --- | --- | --- | --- |
| 20-29 | 2 | <5 | 8 |
| 30-39 | 12 | 5-9 | 7 |
| 40-49 | 3 | 10-19 | 4 |
| 50-59 | 6 | >20 | 12 |
| >60 | 8 |  |  |

**Correction to reference list:**

We noted there was a duplication of a reference and a formatting error in references 16 and 18. We unified the two references and clarified the cited article:

- Albarqouni L, Hoffmann T, Straus S, Olsen NR, Young T, Ilic D, et al. Core Competencies in Evidence-Based Practice for Health Professionals: Consensus Statement Based on a Systematic Review and Delphi Survey. JAMA Network Open. 2018 Jun 22;1(2):e180281.
